# Missing Denture: CT-Negative Radiolucent Non‑clasp Resin Partial Denture Retained in the Stomach for 10 Days

**DOI:** 10.7759/cureus.114466

**Published:** 2026-08-13

**Authors:** Yuji Noda, Kenji Noda, Junichi Shimada, Shinji Yamamoto

**Affiliations:** 1 Emergency Medicine, Asao General Hospital, Kawasaki, JPN; 2 Dentistry, Noda Dental Clinic, Tokyo, JPN; 3 Surgical Gastroenterology, Asao General Hospital, Kawasaki, JPN

**Keywords:** accidental ingestion, ct negative, non-clasp denture, radiolucent object, upper gastrointestinal endoscopy

## Abstract

Accidental denture ingestion in older adults can cause gastrointestinal injury and may be diagnostically challenging when the prosthesis is radiolucent. We report a 76-year-old man who accidentally swallowed a metal-free non-clasp resin partial denture (approximately 1 × 3 cm) while eating. After 10 days, he presented with persistent epigastric pain. Non-contrast chest-abdomen computed tomography (CT) with multiplanar reconstruction (axial, coronal, and sagittal images), interpreted by a board-certified radiologist, showed no definite intraluminal foreign body and no secondary signs of perforation or obstruction. Given the reliable ingestion history and persistent symptoms, urgent esophagogastroduodenoscopy revealed a darkened resin denture in the mid-gastric body along the greater curvature. The denture was retrieved using a retrieval net because net traction alone was insufficient to pass the laryngeal inlet; grasping forceps were used to complete transoral extraction without complications. This case shows that a negative non-contrast CT does not exclude retention of a radiolucent denture, and early endoscopic evaluation should be considered when clinical suspicion remains high.

## Introduction

Accidental ingestion of removable dental prostheses is an important cause of upper gastrointestinal foreign body presentation in older adults. Removable dentures can be broadly classified as complete dentures and removable partial dentures; removable partial dentures may further be designed with conventional metal clasps or with metal-free clasp-retaining components, commonly referred to as non-clasp or flexible dentures. Conventional dentures may contain metal components that facilitate radiographic detection, whereas metal-free acrylic or resin-based dentures may be radiolucent and therefore more difficult to identify on imaging. Diagnosis may be delayed when the prosthesis is radiolucent, particularly for metal-free acrylic/resin dentures, and complications can increase with prolonged retention. Prosthodontic literature has long recognized that conventional denture acrylic resins can be insufficiently radiopaque, motivating development of radiopaque acrylic formulations [1,2]. We report a case of a CT-negative radiolucent denture retained in the stomach for 10 days and removed endoscopically, emphasizing history- and symptom-driven management consistent with guideline recommendations [3]. 

## Case presentation

A 76-year-old man accidentally swallowed a removable partial denture while eating inari sushi. After 10 days, he presented with persistent epigastric pain and concern that the missing denture might have been retained in his gastrointestinal tract.

On arrival, his temperature was 36.5°C. During pre-procedure assessment, vital signs included a pulse of 73/min, a blood pressure of 175/115 mmHg, and an oxygen saturation of 95% on room air. Physical examination revealed mild epigastric tenderness without peritoneal signs. 

**Figure 1:**
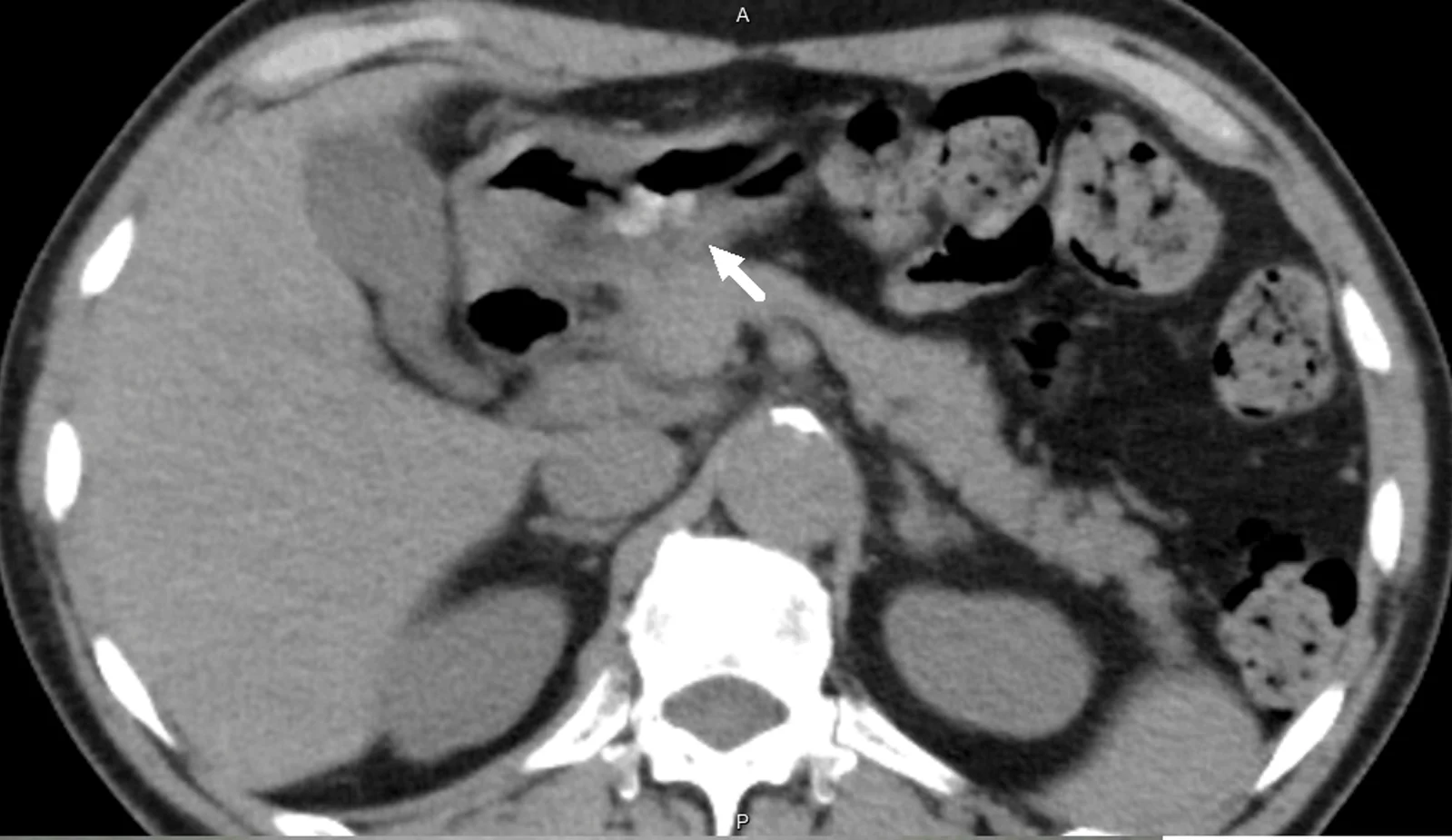
CT Non-contrast chest–abdomen CT showing no definite intraluminal foreign body and no secondary signs of perforation or obstruction. Retrospective radiologic review demonstrated only a subtle low-attenuation structure that could not be confidently distinguished from normal gastric folds or intragastric contents (arrow).

A non-contrast chest-abdomen CT was performed with multiplanar reconstruction (MPR); axial, coronal, and sagittal images were reviewed. The radiologist's report stated that a denture-like foreign body could not be identified, and there were no secondary findings suggestive of gastrointestinal perforation or obstruction (Figure 1).

Despite negative CT findings, we consider his ongoing symptoms 10 days after ingestion; urgent diagnostic/therapeutic EGD was undertaken [1].

**Figure 2:**
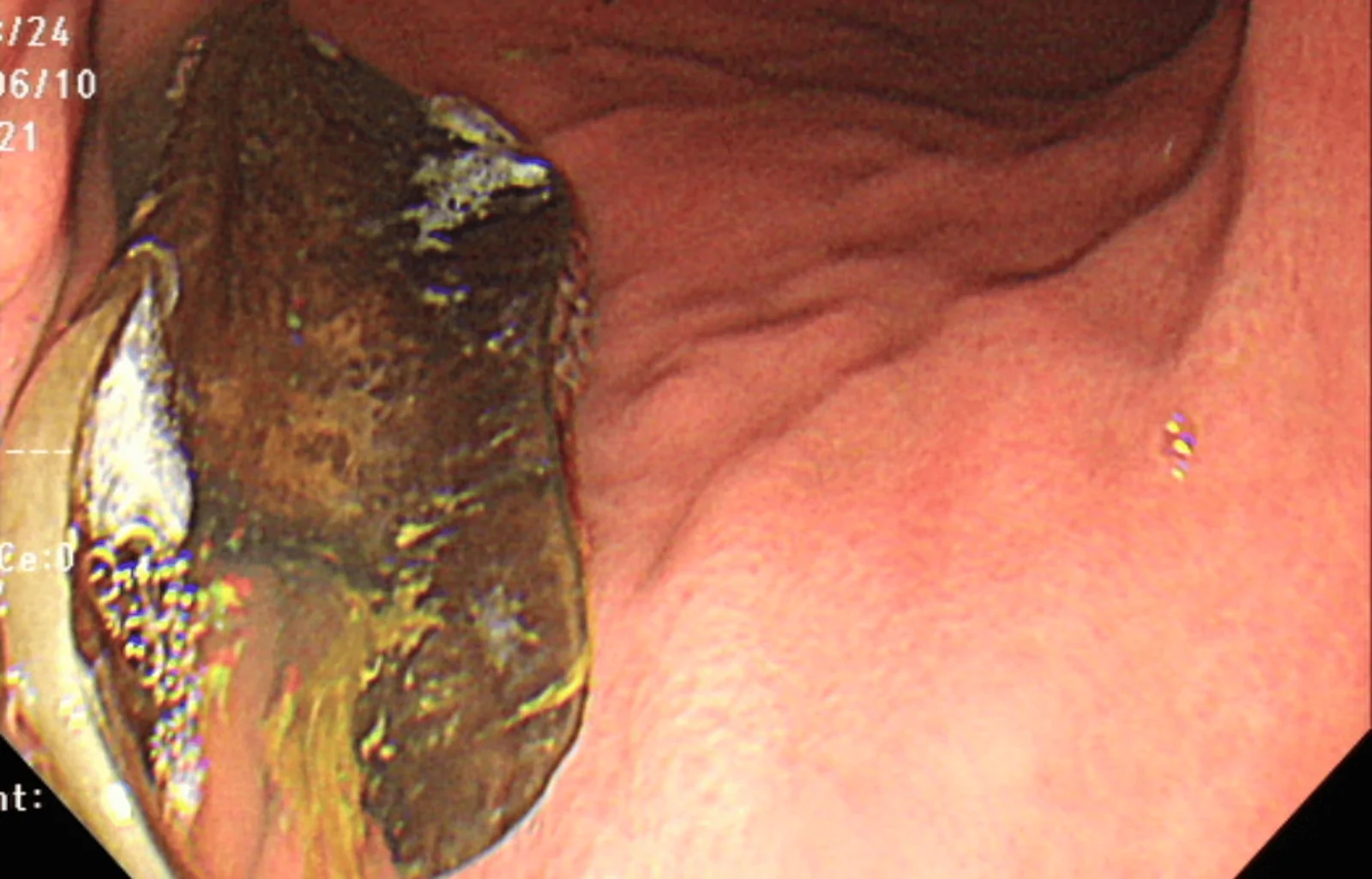
EGD image at gastric body EGD demonstrating a darkened resin partial denture in the mid-gastric body along the greater curvature.

Upper endoscopy was performed with a GIF‑H290 endoscope. The patient requested sedation; intravenous midazolam was administered (0.4 mg initially, then 0.2 mg additional; total 0.6 mg). CO₂ insufflation was used. EGD revealed a denture in the mid-gastric body along the greater curvature, with darkening consistent with exposure to gastric contents (Figure 2). 

**Figure 3:**
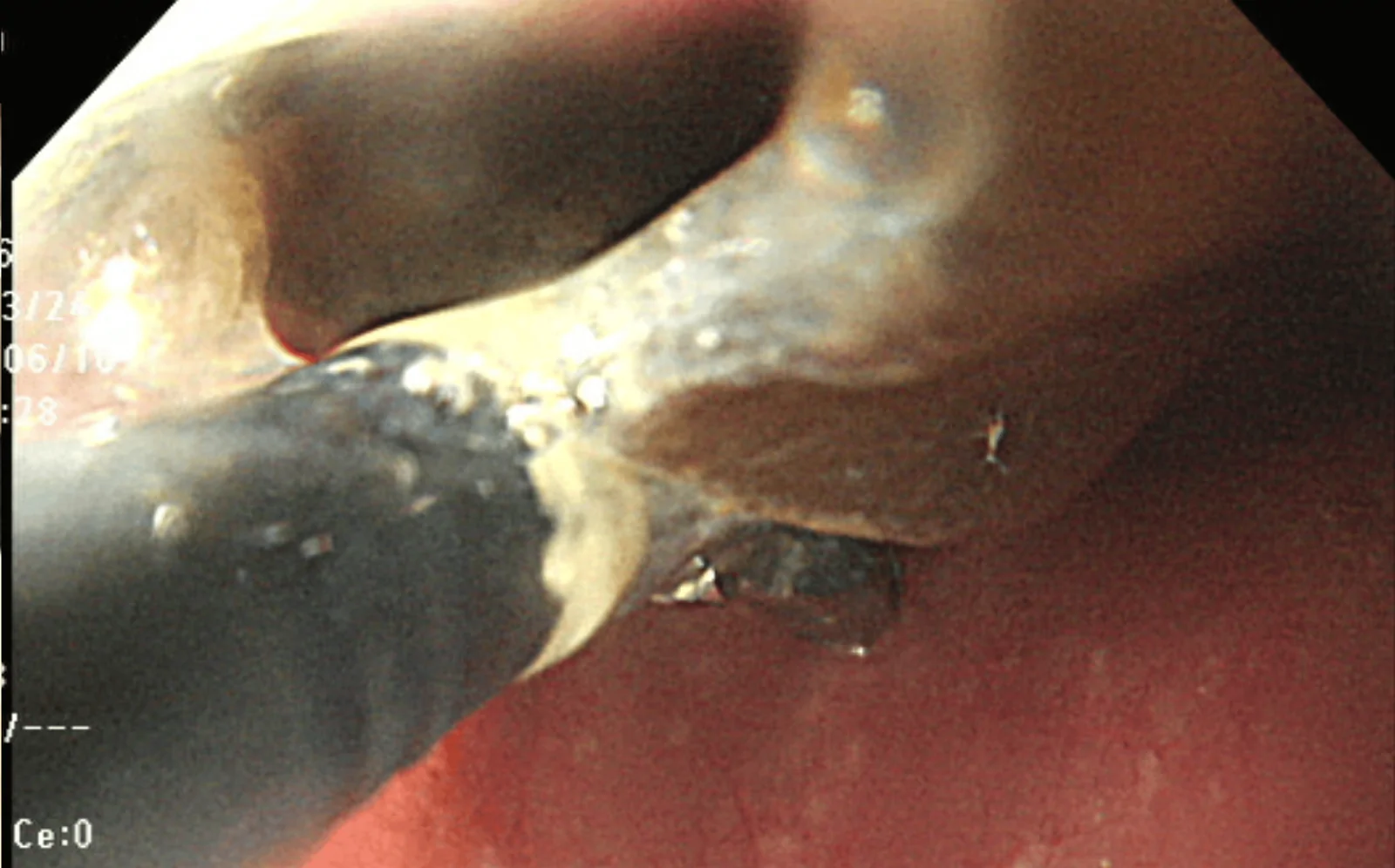
EGD at esophargas Retrieval using a net with subsequent completion of extraction using grasping forceps.

A retrieval net was used to capture the foreign body. During withdrawal, resistance was encountered at the laryngeal inlet, and net traction alone was insufficient to complete transoral extraction. The foreign body was then removed using grasping forceps without complications (Figure 3).

**Figure 4:**
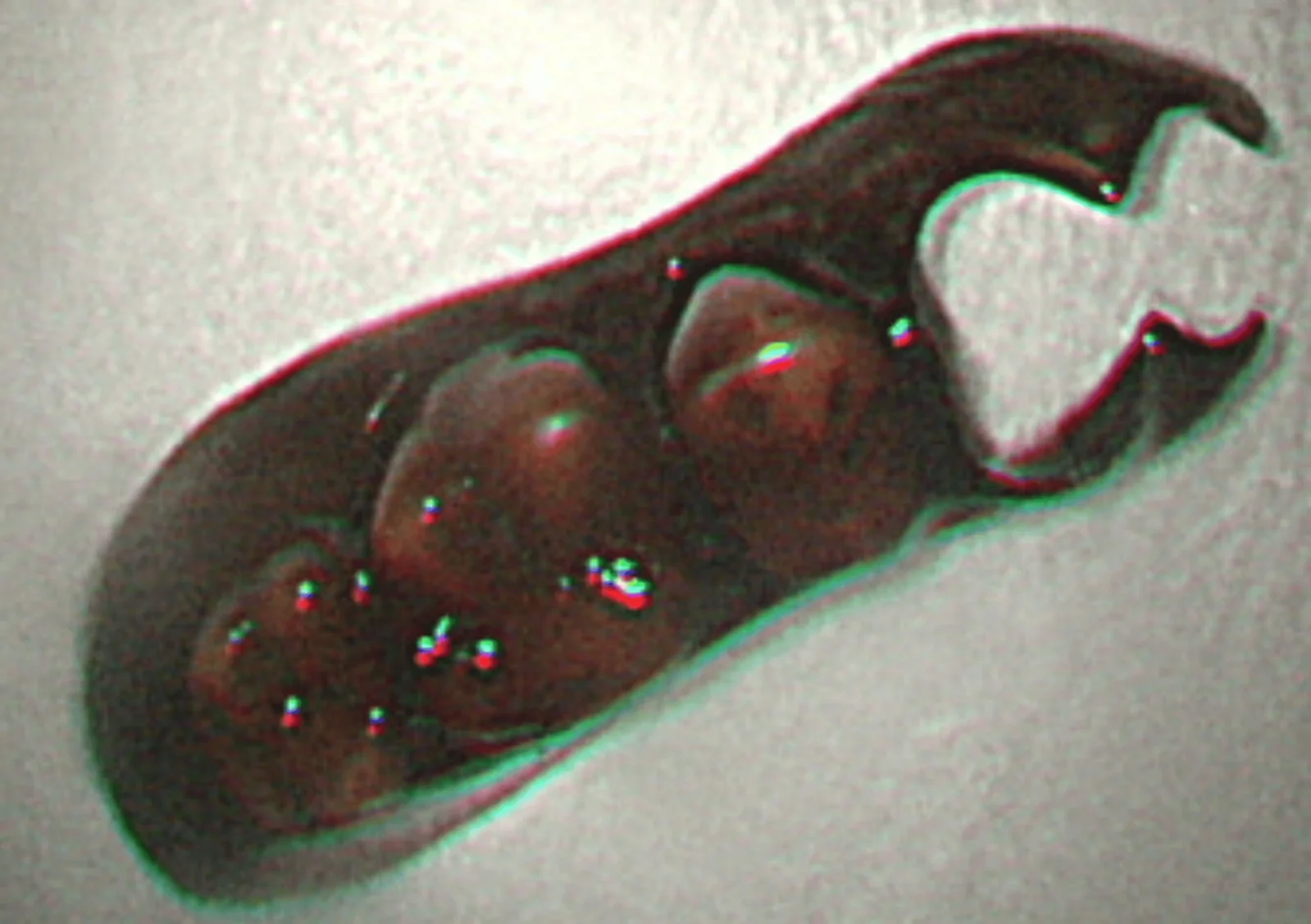
Non-clasp resin partial denture Retrieved metal-free non-clasp resin partial denture (approximately 1 × 3 cm)

Post-extraction endoscopic inspection demonstrated no significant iatrogenic mucosal injury. The retrieved denture was described as a non-clasp resin partial denture without metallic components, approximately 1 × 3 cm in size (Figure 4). 

The patient improved clinically after removal and was managed with return precautions. 

## Discussion

This case demonstrates that a metal-free resin partial denture remained radiographically occult on initial CT. Even after retrospective review with knowledge of the endoscopic findings, only a subtle low-attenuation structure could be identified, remaining difficult to distinguish from gastric folds or intragastric contents.

A major contributor is the intrinsic radiolucency of conventional denture acrylic resins. Bloodworth and Render reviewed the literature and surveyed practitioners regarding acrylic denture radiopacity, highlighting that limited radiopacity is a practical diagnostic problem and that there is longstanding clinical need for radiopaque denture base materials [1]. Mattie and colleagues further support this mechanism by describing the development of a radiopaque autopolymerizing acrylic resin, reflecting that standard acrylic resins may be radiographically occult unless modified [2]. In addition, Newton et al. specifically investigated CT detection of radiolucent denture base material and suggested that CT has advantages over conventional radiography for detecting this material, supporting the rationale for CT use while acknowledging that detectability may still vary by material and imaging conditions [4].

The clinical significance of delayed recognition is reinforced by a 10-year review of published cases of ingested and aspirated dentures, in which Daniels et al. identified complications in a substantial proportion of patients and demonstrated that longer symptom duration and delayed removal were associated with higher complication rates [5]. Although CT can be valuable for evaluating suspected perforation or other complications, this case illustrates that imaging alone may be insufficient for radiolucent prostheses and should not override a high pretest probability based on history and persistent symptoms. Imaging-based characterization of object morphology and location should be integrated with clinical features to guide the need for endoscopic or surgical management [6].

Our management is consistent with European Society of Gastrointestinal Endoscopy (ESGE) recommendations [3]. ESGE emphasizes history- and symptom-driven evaluation for suspected upper gastrointestinal foreign bodies and recommends CT when perforation or other surgical complications are suspected. ESGE also classifies partial dentures as sharp irregular objects and recommends urgent (within 24 hours) therapeutic EGD for sharp-pointed and sharp irregular objects in the stomach if feasible [3]. In the present case, although CT did not identify the foreign body and showed no secondary signs of complication, persistent epigastric pain justified urgent EGD for definitive diagnosis and treatment. Procedure planning should also account for the higher-risk nature of foreign body/impaction endoscopy, including aspiration and perforation considerations [7].

From a technical standpoint, ESGE recommends selection of retrieval devices according to object type/location and use of protective strategies to minimize pharyngo-esophageal injury during extraction of sharp objects [3]. In this patient, a retrieval net enabled secure capture in the stomach, but controlled passage through the laryngeal inlet required transition to grasping forceps to complete safe transoral extraction. Alternative strategies may include direct grasping with forceps, snare-assisted extraction, or the use of an overtube or distal attachment to protect the esophageal and pharyngeal mucosa during withdrawal. An overtube can provide protection during extraction; however, in the present case, we elected not to use an overtube because the relatively irregularly shaped resin denture could potentially become caught within the overtube during withdrawal, making retrieval more difficult. Instead, the denture was carefully captured, and its orientation was controlled under direct endoscopic visualization during transoral extraction. A distal attachment was also not used because the denture could be securely manipulated under direct visualization. These considerations illustrate that the choice of protective devices should be individualized according to the size, shape, flexibility, and material of the retained prosthesis. After successful, uncomplicated removal, discharge is acceptable per ESGE guidance [3].

## Conclusions

Radiolucent metal-free resin partial dentures may be missed on non-contrast CT even with MPR. When ingestion history is reliable and symptoms persist, early EGD should be considered despite negative imaging to confirm the diagnosis and enable safe retrieval. Awareness of the radiolucent nature of these dentures may help prevent delayed diagnosis.
